# Distinct Methylation Changes at the *IGF2-H19* Locus in Congenital Growth Disorders and Cancer

**DOI:** 10.1371/journal.pone.0001849

**Published:** 2008-03-26

**Authors:** Adele Murrell, Yoko Ito, Gaetano Verde, Joanna Huddleston, Kathryn Woodfine, Margherita Cirillo Silengo, Filippo Spreafico, Daniela Perotti, Agostina De Crescenzo, Angela Sparago, Flavia Cerrato, Andrea Riccio

**Affiliations:** 1 Department of Oncology, University of Cambridge, CRUK Cambridge Research Institute, Cambridge, United Kingdom; 2 Dipartimento di Scienze Ambientali, Seconda Università di Napoli, Caserta, Italy; 3 Institute of Genetics and Biophysics “A. Buzzati Traverso”, Consiglio Nazionale delle Ricerche (CNR), Naples, Italy; 4 Dipartimento di Scienze Pediatriche e dell'Adolescenza, Università di Torino, Torino, Italy; 5 Pediatric Oncology Unit, Department of Medical Oncology, Fondazione IRCCS Istituto Nazionale dei Tumori, Milano, Italy; 6 Department of Experimental Oncology and Laboratories, Fondazione IRCCS Istituto Nazionale dei Tumori, Milano, Italy; Fred Hutchinson Cancer Research Center, United States of America

## Abstract

**Background:**

Differentially methylated regions (DMRs) are associated with many imprinted genes. In mice methylation at a DMR upstream of the *H19* gene known as the Imprint Control region (IC1) is acquired in the male germline and influences the methylation status of DMRs 100 kb away in the adjacent Insulin-like growth factor 2 (*Igf2)* gene through long-range interactions. In humans, germline-derived or post-zygotically acquired imprinting defects at IC1 are associated with aberrant activation or repression of *IGF2*, resulting in the congenital growth disorders Beckwith-Wiedemann (BWS) and Silver-Russell (SRS) syndromes, respectively. In Wilms tumour and colorectal cancer, biallelic expression of *IGF2* has been observed in association with loss of methylation at a DMR in *IGF2.* This DMR, known as DMR0, has been shown to be methylated on the silent maternal *IGF2* allele presumably with a role in repression. The effect of *IGF2* DMR0 methylation changes in the aetiology of BWS or SRS is unknown.

**Methodology/Principal Findings:**

We analysed the methylation status of the DMR0 in BWS, SRS and Wilms tumour patients by conventional bisulphite sequencing and pyrosequencing. We show here that, contrary to previous reports, the *IGF2* DMR0 is actually methylated on the active paternal allele in peripheral blood and kidney. This is similar to the IC1 methylation status and is inconsistent with the proposed silencing function of the maternal *IGF2* allele. Beckwith-Wiedemann and Silver-Russell patients with IC1 methylation defects have similar methylation defects at the *IGF2* DMR0, consistent with IC1 regulating methylation at *IGF2* in *cis*. In Wilms tumour, however, methylation profiles of IC1 and *IGF2* DMR0 are indicative of methylation changes occurring on both parental alleles rather than in *cis*.

**Conclusions/Significance:**

These results support a model in which DMR0 and IC1 have opposite susceptibilities to global hyper and hypomethylation during tumorigenesis independent of the parent of origin imprint. In contrast, during embryogenesis DMR0 is methylated or demethylated according to the germline methylation imprint at the IC1, indicating different mechanisms of imprinting loss in neoplastic and non-neoplastic cells.

## Introduction

Aberrant imprinting of the Insulin-like growth factor 2 (*IGF2*) gene plays a role in the pathogenesis of the overgrowth disorder Beckwith-Wiedemann syndrome (BWS, OMIM#130650), the growth-restriction condition Silver-Russell syndrome (SRS, OMIM#180860), as well as various human cancers including Wilms tumour, rhabdomyosarcoma, hepatoblastoma, colorectal and breast carcinomas [1]–[6].

Mouse models have been used to demonstrate that the imprinted expression of the closely linked *Igf2* and *H19* genes is controlled by specific differentially methylated regions (DMRs) [7]–[10]. One of these DMRs (the *H19* DMR) is located 5′ of the *H19* promoter is methylated on the paternal chromosome and is known as the imprinting control region IC1 because it functions as an insulator mediated by the CCCTC binding factor (CTCF) [11]–[14]. Deletion of the maternal IC1 results in activation of the normally silent maternal *Igf2* allele and in down-regulation of *H19* [9]. In addition, a hierarchical relationship between methylation at IC1 and *Igf2* exists such that deletion or mutation within the IC1 also results in methylation changes in DMRs within the *Igf2* gene [15]. We have previously shown that IC1 actually interacts with *Igf2* DMRs in a mutually exclusive and parent of origin specific manner influenced by CTCF binding and DNA methylation [16], [17]. The application of this model to the human has been hampered by differences in the genomic organisation of *IGF2* DMRs in mouse and human. Specifically, *IGF2* DMR0, located 5′ to the main *IGF2* promoters is methylated on the maternal allele only in placentas in the mouse [18], but has differential methylation in all tissues in humans [19]. Methylation of the DMR0 on the maternal *IGF2* allele was inferred using cell lines derived from a BWS patient with paternal uniparental disomy at 11p15.5 loci (UPD) [19] and in Wilms tumour kidney patients with loss of heterozygosity of the maternal allele [20]. Methylation analysis of the *IGF2* DMR0 has been carried out in many cancer studies following the demonstration that hypomethylation of this DMR is associated with loss of imprinting in Wilms tumour [20] and colorectal cancer [21]. The current interpretation of these results is that loss of methylation at the *IGF2* DMR0 plays a role in reactivation of the silent maternal allele [21].

The molecular basis of BWS is heterogeneous with 5% of patients exhibiting gain of methylation at IC1 with biallelic activation of *IGF2* and biallelic silencing of *H19.* Another group of BWS patients has normal IC1 methylation but abnormal methylation at IC2, a maternally methylated imprinting control region within the *KCNQ1* and *KCNQ1OT1* gene cluster which seems to operate independent of *IGF2* and *H19* [2]. BWS patients with IC1 defects have a particularly high predisposition to developing Wilms tumour, while those patients with IC2 defects are apparently more at risk for other tumours [2], [22]. Hypermethylation at the IC1 is also a frequent feature in non-syndromic Wilms tumour patients which together with the distinct tumour predisposition profiles in BWS patients suggest that similar epigenetic defects lead to soma-wide overgrowth and cancer. SRS also has a complex heterogeneous molecular aetiology and a subset of individuals has loss of methylation at IC1 with biallelic silencing of *IGF2* and biallelic activation of *H19* [6].

To date there have been no reports on the methylation status of the *IGF2* DMR0 region in BWS and SRS patients and it is not known to what extent the proposed silencing function of this DMR contributes to the aetiology of BWS and SRS. We therefore examined parent of origin methylation at the DMR0 in various subtypes of BWS and SRS patients and also in a cohort of Wilms tumour samples. Our results indicate that the active paternal *IGF2* allele is methylated at the DMR0 in *cis* with *H19* methylation in normal individuals and that in SRS and BWS the DMR0 and IC1 have similar methylation abnormalities. In contrast, in Wilms tumour samples DMR0 methylation is negatively correlated with IC1 methylation. These results imply *IGF2* imprinting defects in congenital growth disorders and Wilms tumours arise through different epigenetic mechanisms.

## Results

### IGF2 DMR0 methylation status in BWS and SRS patients

**Figure 1:**
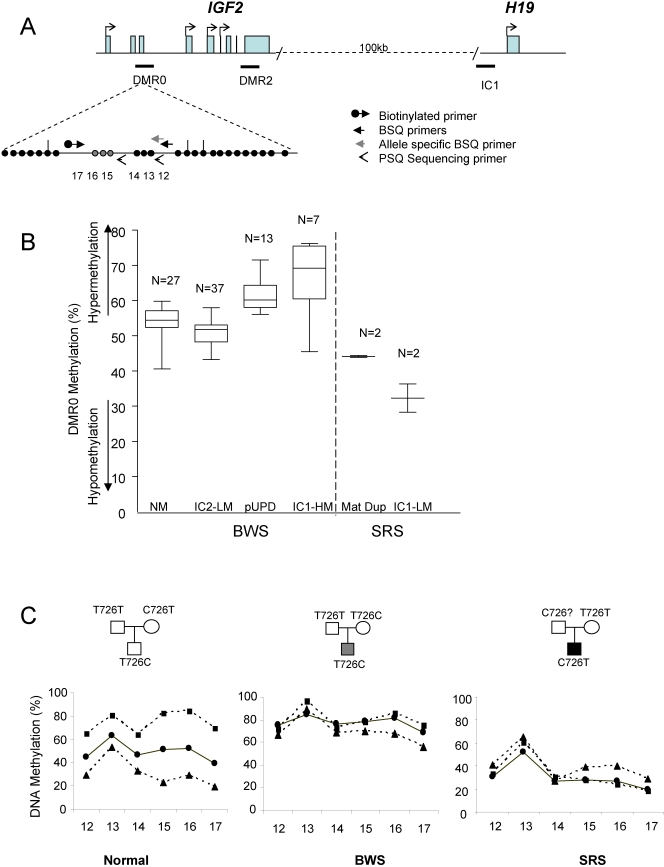
IGF2 DMR0 Methylation levels in Beckwith-Wiedemann Syndrome (BWS) and Silver-Russell Syndrome (SRS) patients. A. Location of DMR0 within the *IGF2* gene relative to the paternally methylated imprinting control region IC1 which is upstream of *H19.* Pyrosequencing was used to analyse methylation at six CpGs within the DMR0 region (NCBI 36: 11, 2125904–2126160, containing SNP rs3741210). Bisulphite (BSQ) and pyrosequencing (PSQ) primer positions are as indicated. This region contains the CpGs (numbered 15,16,17) marked in grey which have previously been published to be hypomethylated in Colorectal cancer [21]. Vertical lines indicate *Msp*I/*Hpa*II sites in the DMR0. B. Box plot showing medians, IQR, max. and min. values for *IGF2* DMR0 methylation levels as measured by pyrosequencing of bisulphite converted DNA obtained from peripheral blood samples from BWS and SRS patients. Methylation percentages reflect the proportion of bisulphite converted cytosines at the CpGs measured in the assay. Normal methylation levels for imprinted DMRs are 45–55% (see C). Categories of BWS patients included those with: no imprinting defect at the IC1 region and the IC2 region (NM); low methylation (LM) at the IC2 (IC2-LM); increased paternal 11p15 through paternal duplication or uniparental disomy (pUPD); and hypermethylation at IC1 (IC1-HM). SRS patients included two each of maternal duplication of 11p15 (Mat Dup) and hypomethylation at IC1 (IC1-LM). BWS with hypermethylation at the IC1, also have hypermethylation at DMR0, while SRS with hypomethylation IC1 have hypomethylation at DMR0. C. Parent of origin specific methylation in normal, BWS and SRS patients. The BWS and SRS patients have imprinting defects at IC1. Bisulphite converted DNA was amplified with allele-specific primers for the rs3741210 polymorphism in *IGF2* DMR0 prior to pyrosequencing for methylation. The T allele is shown by squares and C alleles shown by triangles in the plots. Circles show the percentage of total methylation of the CpGs assayed. The paternal allele is more methylated at DMR0 than the maternal allele. Both alleles are hypermethylated in BWS and both alleles are hypomethylated in SRS.

We examined the methylation status of DMR0 in peripheral blood leukocyte DNA derived from BWS patients with IC1 hypermethylation (n =  7), IC2 hypomethylation (n = 37) or normal methylation at both IC1 and IC2 (n = 27) and SRS patients with IC1 hypomethylation (n =  2), using bisulphite and pyrosequencing analysis. The region analysed is shown in figure 1A. To our surprise, 6/7 BWS patients with hypermethylation at IC1 also had hypermethylation at DMR0 (Median methylation 69.2%, interquartile range (IQR) 60.5%; 75.1%) and 2/2 SRS with IC1 hypomethylation also had DMR0 hypomethylation (28.2% and 36.28%). Patients with normal methylation at IC1 or hypomethylation at IC2 had normal methylation levels at DMR0 (Median methylation 53%; IQR 48.2%, 57.1%). These results suggest that either methylation changes in BWS and SRS were occurring in *trans* or that methylation at the DMR0 is on the paternal allele.

To distinguish between these possibilities, we examined 13 BWS patients with excess copies of paternal 11p15.5 (12 patients had paternal UPD and one patient had an 11p15.5 duplication) and two SRS patients with maternal 11p15.5 duplications. We also determined directly the parental origin of methylation at the *IGF2* DMR0 in some of our BWS and SRS patients and 6 control individuals in order to confirm our findings. The patients with maternal duplication had reduced methylation levels (44%), while all the paternal UPD and the paternal duplication patients had hypermethylation ((Median methylation 60.2%; IQR 56.1%, 64.3%), Fig. 1B). In all informative families we were able to confirm that the paternal allele was preferentially methylated at the DMR0 (particularly the CpGs 15–17) in *cis* with methylation at the IC1 (Fig. 1C and Fig. S1). These results therefore suggest that methylation at the IC1 influences methylation at the *IGF2* DMR0. BWS and SRS cases with methylation defects at the IC1 acquire the same methylation defect at the DMR0 in the germline or early fetal development such that the maternal *IGF2* allele gains methylation and is activated (BWS) or the paternal allele fails to become methylated and is silenced (SRS). These allele-specific methylation changes have the effect of an imprint switch so that both alleles look like either a paternal allele (BWS) or a maternal allele (SRS). Pyrosequencing results were verified by bisulphite sequencing (Fig. S1).

### IGF2 methylation analysis in Wilms Tumour samples

**Figure 2:**
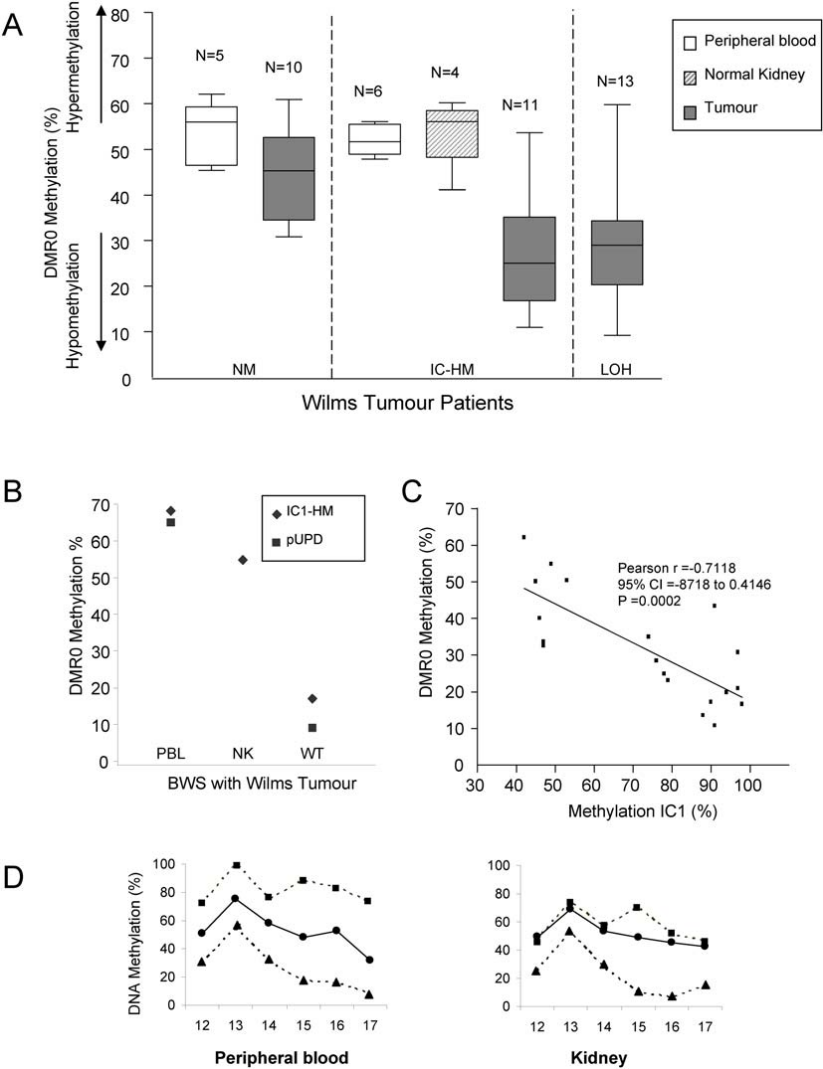
Methylation levels at the IGF2 DMR0 region in Wilms tumour patients. A. Box plot showing medians, IQR, max. and min. values for *IGF2* DMR0 methylation levels as measured by pyrosequencing of peripheral blood (open box), normal kidney (hatched box) and tumour biopsies (grey box) from Wilms Tumour patients that do not have BWS. Methylation percentages reflect the proportion of bisulphite converted cytosines at the CpGs measured in the assay. Normal methylation levels for imprinted DMRs are 45–55% (see D). Wilms Tumour patient samples include peripheral blood and tumour DNA from patients with normal methylation at IC1 in their tumours (NM); peripheral blood, normal kidney and tumour DNA samples from patients with hypomethylation of IC1 in their tumours (IC1-HM); and tumour DNA samples from patients with loss of heterozygosity (LOH) in their tumours. Strikingly, Wilms Tumour patients with hypermethylation at IC1 have hypomethylation at DMR0 in their tumours. B Methylation levels in peripheral blood, unaffected kidney tissue and Wilms tumour kidney tissue from two BWS patients (one with IC1 hypermethylation and one with pUPD). Both patients have hypomethylation of *IGF2* DMR0 in their tumours. C Linear regression curve showing inverse relationship between methylation levels at the IC1 and DMR0 in Wilms Tumour. Tumours with hypermethylation at the IC1 have loss of methylation at DMR0. D. Peripheral blood and kidney tissue from a patient with Wilms Tumour that does not have hypermethylation at IC1. The C-allele is hypermethylated, while the T-allele is hypomethylated in both the blood and kidney–indicating similar parent of origin methylation in these tissues.

We analysed DMR0 methylation in two Wilms tumour patients that had arisen in individuals affected by BWS and a series of non-syndromic Wilms tumour patients. One of the BWS patients had constitutional hypermethylation at IC1, the other had paternal UPD. The non-syndromic Wilms tumour patients comprised of individuals that had tumour-specific IC1 hypermethylation (n = 10), 11p15.5 LOH with loss of the maternal allele (n = 13) or normal methylation (n = 10) at the IC1. Remarkably, all tumours with hypermethylation at IC1 and most of those with LOH, including those arisen in individuals affected by BWS, showed reduced methylation at DMR0 (Median methylation 24.8%, IQR 16.52, 34,9), while those with normal IC1 methylation had normal DMR0 methylation (Median methylation 45.1%, IQR 34.2; 52.3) (Fig. 2A–B). Indeed, a significant inverse correlation (Pearson r = 0.7118, CI -8718-0.4146) could be shown between DMR0 and IC1 methylation levels in Wilms tumour patient (Fig. 2C). As in blood leukocytes, adjacent non tumour tissue showed predominant methylation of the paternal allele (Fig. 2D and data not shown). These results show distinct epigenetic changes in Wilms tumour compared to congenital growth disorders.

## Discussion

The results of our genetic studies demonstrate that DMR0 is methylated on the paternal allele contradicting the conclusions of two previous studies which have reported that DMR0 methylation is on the maternal allele [19], [20]. In the first report methylation was investigated for the entire *IGF2* gene in Wilms tumour samples with and without loss of imprinting [20]. In this study, differential methylation at the DMR0 region was shown in normal kidney and in tumours with monoallelic expression, whereas tumours with loss of imprinting, were hypomethylated [20]. The CpGs that we analysed in our methylation assays were also examined by these authors using *Msp*I*/ Hpa*II restriction analysis (Figure 1) and the region is spanned by probe 9 in figures 1 and 3 in [20]. In four cases with loss of heterozygosity of 11p15.5 where the maternal allele was lost, the retained paternal allele was found to be unmethylated and thus the maternal allele was inferred to be the normally methylated allele [20]. We examined 13 cases with LOH and showed similar hypomethylation patterns, which suggests that cells with LOH are inclined to methylation changes at the DMR0 as a result of being tumours. The second study to report that DMR0 is maternally methylated characterised novel transcripts of *IGF2* during development. These authors examined a cell line established from pUPD patient and found that the DMR0 was hypomethylated [19]. We have found that DMR0 tends to be demethylated in hybridoma cell lines with a single human chromosome 11, regardless of parental origin (unpublished observation) and assume that this may be a cell culture phenomenon.

The function of DMR0 is still unknown. It has been postulated that the role of DMR0 methylation is to maintain silencing on the maternal *IGF2* allele. However, the knowledge that the parental origin of *IGF2* DMR0 methylation is paternal eliminates a role in methylation-mediated repression of the maternal *IGF2* allele. Indeed rather than acting as an autonomous regulator of imprinting, our results suggest that the DMR0 methylation is influenced by IC1 methylation in non-neoplastic cells. Thus during embryogenesis, parent of origin specific methylation patterns at the human *IGF2*-*H19* locus are established in *cis* similar to observations in mice [15].

**Figure 3:**
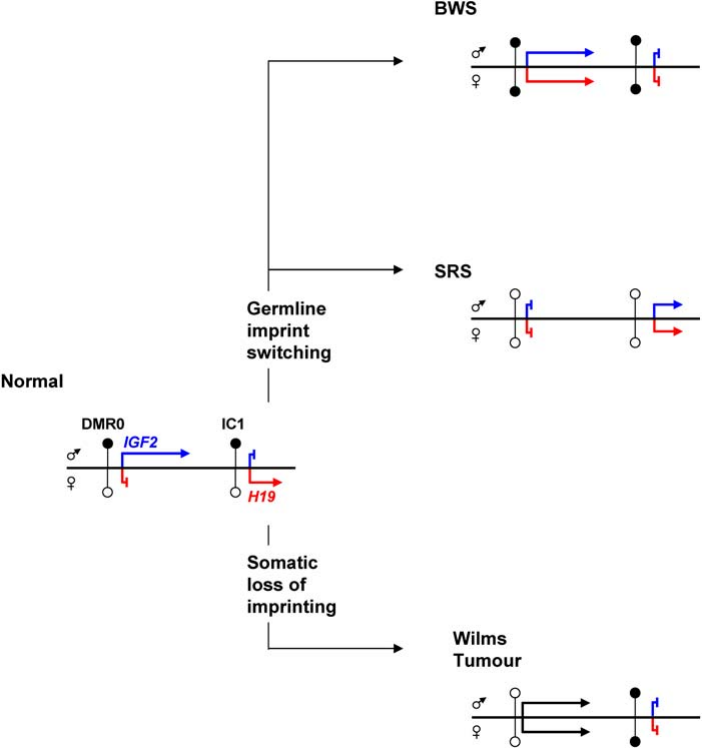
Summary of IGF2 DMR0 Methylation relative to IC1 in normal, congenital growth disorders and Wilms tumour. In normal individuals, *IGF2* expression is from the paternal allele and *H19* is from the maternal allele. IC1 and DMR0 are methylated on the paternal chromosome. In BWS, the epigenotype and expression pattern of the maternal chromosome are converted to paternal and in SRS, the epigenotype and expression pattern of the paternal chromosome are converted to maternal (imprint switching). In the Wilms tumour, *IGF2* activation and *H19* silencing are associated with disruption of both maternal and paternal imprints (somatic loss of imprinting).

In mice where CTCF sites have been mutated, the exclusion of CTCF from the maternal IC1 has resulted in methylation of the IC1 [23] and *Igf2* DMRs [16]. Furthermore, in adult mouse choroid plexus, a brain tissue in which *Igf2* is expressed from both alleles and *H19* is not expressed, IC1 and the *Igf2* DMRs are methylated on both parental chromosomes [24]. We show here that aberrant gain or loss of methylation at the IC1 is coupled to the same methylation change at *IGF2* DMR0. The main difference is that mice have a DMR1 and a placenta specific DMR0, whereas in humans, DMR1 is lacking and the DMR0 seems to behave more similar to mouse DMR1. The ultimate effect of IC1 influencing *IGF2* methylation is that epimutation of IC1 has the consequence of both parental alleles displaying a paternal imprint in the case of BWS or a maternal imprint in the case of SRS (Fig. 3).

If long-range interactions between DMRs at this locus are similar in mice and humans, this would explain the imprinting defects that are found in the overgrowth disorders BWS and SRS. However, the imprinting defects in Wilms tumour remain difficult to reconcile with enhancer competition and long-range DMR interaction models. In our analysis of this selected group of Wilms tumour patients, methylation at the DMR0 is lost on the paternal allele, while IC1 gains methylation on the maternal allele. DMR0 methylation levels are particularly low in some of the tumours, indicating an almost complete eradication of methylation at this site probably from both alleles (Fig. 2A–C). Epigenetic reprogramming thus occurs on both parental chromosomes rather than in *cis*, indicating that different mechanisms control *IGF2* imprinting in neoplastic and non-neoplastic cells (Fig. 3). Soma-wide IC1 hypermethylation in BWS predisposes to Wilms tumour and similar epigenetic changes occurs in the kidney as pre-neoplastic events in Wilms tumorigenesis [25]. It is possible that DMR0 hypomethylation is associated with a more sustained activation of *IGF2* in tumour cells as a result of alternative high level chromatin conformation promoted by tumour-specific activators.

During tumorigenesis global hypomethylation occurs in repeat rich regions, LINE and SINE elements, while CpG islands associated with promoters are subject to hypermethylation [26]. DMRs methylated in the germline and DMRs methylated in the somatic cells may differ in their ability to be reprogrammed during tumorigenesis. Further comparison of different DMRs in terms of germline versus somatic and maternally versus paternally methylated as well as genetic and epigenetic characteristics should lead to deeper understanding of epigenetic reprogramming in cancer. Prior to our studies, loss of methylation is the only described epimutation at the DMR0 in cancers, while both hyper and hypomethylation has been described at the IC1 [21], [27]. Moreover, not all reports show a direct correlation between *IGF2* expression and DMR0 methylation changes at this locus and we have reported exceptions in breast cancer (Ito *et al.* submitted), while others have reported exceptions in ovarian and bladder cancer [28], [29]. Those studies that do show a relationship between loss of DMR0 methylation and loss of imprinting need to be reinterpreted because the methylation at the DMR0 region is lost from the active paternal allele rather than from the silent allele.

In summary, we show three different reprogramming events occurring at the *IGF2-H19* locus and associated with imprinting loss in BWS, SRS and Wilms Tumour. In the growth disorders, germline allele-specific methylation changes have the effect of an imprint switch so that both alleles look like either a paternal allele (BWS) or a maternal allele (SRS) while in cancer both parental marks are lost and reprogrammed. Our findings demonstrate that loss of *IGF2* imprinting is a complex phenomenon that occurs with different mechanisms in human disease, probably reflecting different molecular causes and responses.

## Materials and Methods

### Subjects

85 BWS and 3 SRS cases were recruited from different Italian Pediatric Departments and were clinically diagnosed according to the criteria described in the literature (http://www.geneclinics.org). The study also involved 40 Wilms Tumour patients enrolled by Paediatric Oncology Units affiliated to Associazione Italiana Ematologia Oncologia Pediatrica (AIEOP). All tumours were histologically diagnosed.

### Ethical Approval

This study was approved by the ethical committees of the Second University of Naples and Istituto Nazionale Tumori, INT, Milan .

### Identification of IC1 and IC2 methylation, UPD, LOH and copy number abnormalities

The DNA methylation at IC1 and IC2 was analysed by Southern blotting with methylation-sensitive restriction enzymes or COBRA, as described [2]. Three samples with IC1 hypermethylation derived from BWS patients also had inherited microdeletions [30], [31]. UPD, LOH and duplication at 11p15.5 loci were determined by microsatellite analysis, as described [32].

### Analyses of DMR0 methylation

We designed a standard pyrosequencing assay for *IGF2* DMR0 region (NCBI36:11,2125904-2126160) which included six CpGs, three of which were previously reported to be hypomethylated in colorectal patients with LOI at *IGF2* [21]. The CpGs within this region are included in the DMR0 region analysed by others [19], [20]. 100 ng–2 ug of genomic DNA per sample was bisulphite treated using EZ DNA methylation kit (Zymo Research). Bisulphite treated DNA was used for generating PCR amplified templates for pyrosequencing using the following forward primer: 5′TGAGGATGGGTTTTTGTTTGGTAT3′ and biotinylated reverse primer 5′TCCTCAATCCACCCAAAATAATAT3′. 10 uL of the biotinylated PCR product was used for each sequencing assay using the following sequencing primers 5′GGGGTGGAGGGTGTA 3′ and 5′-AAAAGTTATTGGATATATAGT 3′. Pyrosequencing was carried on PSQ HS 96 System and PyroMark MD System using Pyro Gold Reagent kits (Biotage, Uppsala, Sweden). Allele-specific pyrosequencing was carried out by replacing the above biotinylated primer with allele-specific biotinylated primers, 5′CCCAAAATAATATCTATAAAAAAAAAATTCAC3′, or 5′CCCAAAATAATATCTATAAAAAAAAAATTCAT3′ which recognized the G or A allele of the rs3741210 polymorphism on the reverse strand after bisulphite conversion.

Methylation was quantified using Pyro Q-CpG Software (Biotage, Uppsala, Sweden) that calculates the ratio of converted C's (T's) to unconverted C's at each CpG and expresses this as a percentage methylation. Average methylation across the DMR0 for all 6 CpGs were calculated. Median and IQR methylation for different categories of patients were analysed using Prism Graphpad software.

We verified allele-specific methylation by conventional bisulphite sequencing of cloned PCR products in all our informative cases.

## Supporting Information

Figure S1 Bisulphite sequencing analyses of IGF2 DMR0 Methylation in normal, congenital growth disorders and Wilms Tumour. IGF2 DMR0 Methylation in normal, congenital growth disorders and Wilms Tumour. Methylation of 6 CpGs was determined by bisulphite genomic sequencing on DNA extracted from peripheral blood leukocytes (Normal, BWS and SRS) or tumour tissue (Wilms Tumour) of individuals informative for the rs3741210 polymorphism. Filled circles represent methylated CpGs and open circles unmethylated CpGs. The maternal (MAT) and paternal (PAT) alleles of the IGF2 DMR0 region are indicated.
(1.33 MB TIF)
