# Supplementary figures and images for: Distinct Methylation Changes at the IGF2-H19 Locus in Congenital Growth Disorders and Cancer
Source: PLoS One. 2008 Mar 26;3(3):e1849. doi: 10.1371/journal.pone.0001849 (PMC2268001; doi:10.1371/journal.pone.0001849)

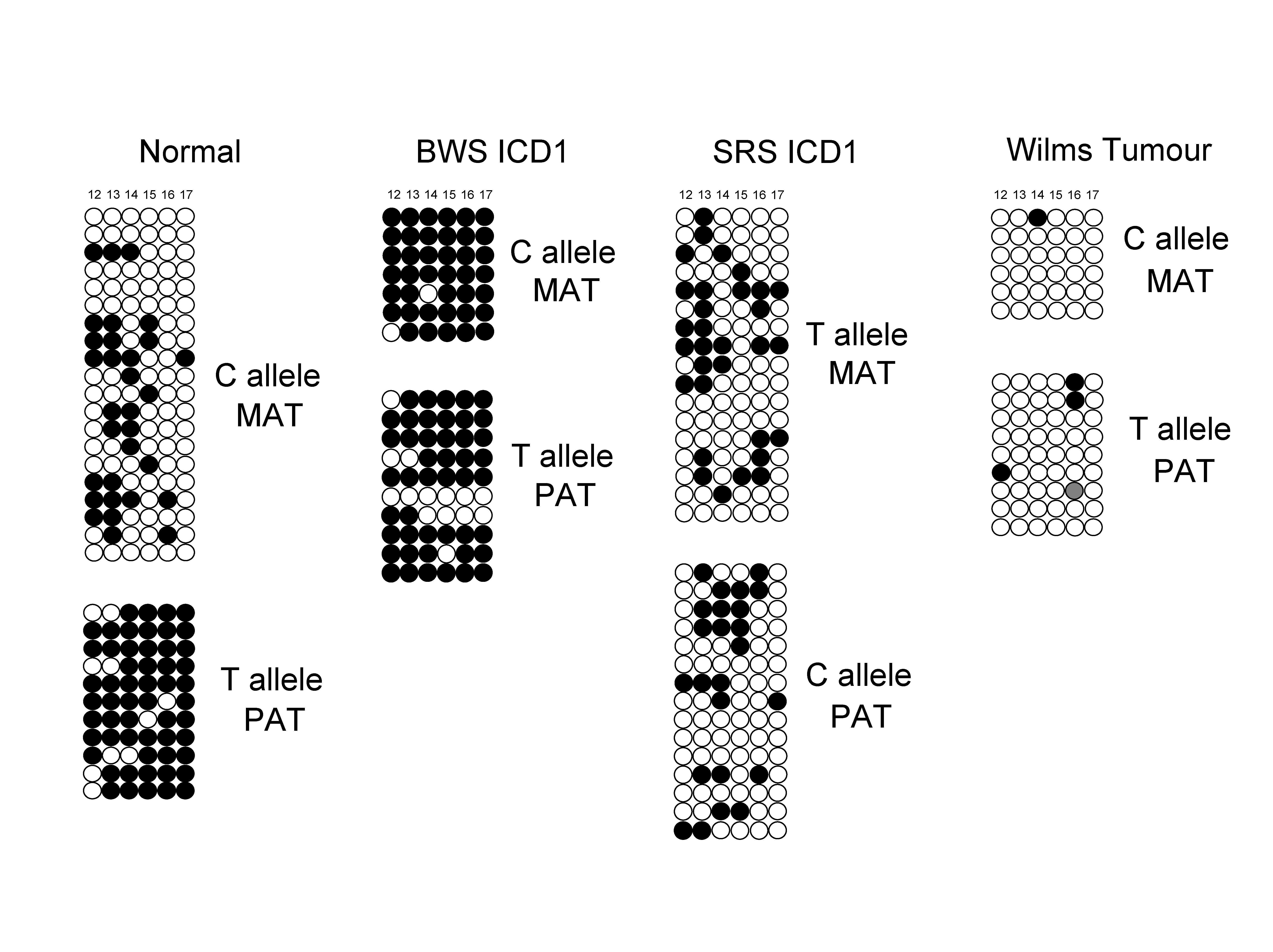

Supplement: Figure S1 — Bisulphite sequencing analyses of IGF2 DMR0 Methylation in normal, congenital growth disorders and Wilms Tumour. IGF2 DMR0 Methylation in normal, congenital growth disorders and Wilms Tumour. Methylation of 6 CpGs was determined by bisulphite genomic sequencing on DNA extracted from peripheral blood leukocytes (Normal, BWS and SRS) or tumour tissue (Wilms Tumour) of individuals informative for the rs3741210 polymorphism. Filled circles represent methylated CpGs and open circles unmethylated CpGs. The maternal (MAT) and paternal (PAT) alleles of the IGF2 DMR0 region are indicated. (1.33 MB TIF) [file pone.0001849.s001.tif]
